# Morality extracted under crowding impairs face identification

**DOI:** 10.1177/20416695221104843

**Published:** 2022-06-24

**Authors:** Risako Shirai, Hirokazu Ogawa

**Affiliations:** 13148 Waseda University, Japan; Japan Society for the Promotion of Science, Japan; Kwansei Gakuin University, Japan

**Keywords:** morality, crowding, visual awareness

## Abstract

We investigated whether morality associated with faces is perceptible even under less optimal visual conditions such as crowding. A facial image was paired with a sentence describing an immoral act or a neutral act. Participants imagined the person performing the actions described in the sentence during the learning phase. Then, in the crowding phase, the target face was briefly presented in the left or right peripheral visual fields. Participants were required to judge the gender or morality of the target face in Experiment 1 and to choose the target face from two faces in Experiment 2. In both experiments, flankers were presented around the target face in the flanker condition, whereas no flankers were presented in the no-flanker condition. Experiment 1 indicated that the accuracy of judgments about the morality of a crowded face was higher for immoral faces than for neutral faces. This demonstrates that morality is preferentially extracted even when conscious access to facial representations is limited. Experiment 2 showed that the accuracy of selecting the flanked face from two faces was higher for neutral faces than for immoral faces. These indicated that the morality processed under the crowding impaired the discrimination of the facial identity.

## Introduction

During the past few decades, moral psychologists have proposed that conscious reasoning is a core process in making moral judgments (Colby et al., 1983; Piaget, 1932/1997; Turiel, 1983). However, recent studies have suggested that intuitions, including emotional valence, rather than conscious reasoning, play a crucial role in moral judgments (Haidt, 2001). Unconscious emotional intuitions are induced when we judge the morality of an episode described by a sentence depicting an offensive act: such as “He cooked and ate his dead pet,” which might contribute to making moral judgments about the episode. Also, from a socio-ecological point of view, promptly evaluating immoral acts such as deception and harming others is crucial for protecting the social order. How Immoral signals affect unconscious processing essential for making quick decisions must be clarified to understand the mechanisms of unconscious and intuitive moral judgments.

Research has examined whether and how morality modulates initial percepts (Anderson et al., 2011; De Freitas & Alvarez, 2018; Gantman & Van Bavel, 2014), although this critical issue remains unsettled. For example, Gantman and Bavel (2014) used word stimuli and demonstrated that morally-relevant words, such as kill, moral, and should, were identified more frequently than morally irrelevant words, such as die, useful, or could, even when the words were presented for a duration that was too short to be perceived. Gantman and Van Bavel interpreted this result as suggesting that morality facilitates awareness of ambiguous stimuli. Moreover, Anderson et al. (2011) used a binocular-rivalry method in which an image of a face was presented to one eye and an image of a house to the other eye and examined how moral information affected conscious awareness. They associated faces with moral information by associative conditioning and demonstrated that faces associated with negative gossip were dominant for longer than faces associated with positive or neutral gossip. However, Stein et al. (2017) examined whether faces associated with negative gossip affected unconscious processing by using the breaking continuous flash suppression (b-CFS) paradigm, which is a technique for suppressing the conscious percept of visual stimuli (Jiang et al., 2007; Stein et al., 2011; Tsuchiya & Koch, 2005). They reported no impact of negative gossip on suppression time and concluded it unlikely that faces associated with negative gossip are prioritized in unconscious processing (see also Rabovsky et al., 2016).

The current study investigated whether the morality of faces modulated the initial perception of faces, even when a face could not be consciously discriminated, by using a visual crowding paradigm to examine unconscious processing. Visual crowding is known to hinder the identification of peripheral objects that are surrounded by similar objects (Levi, 2008). Most theories explaining crowding have proposed that crowding reflects a bottleneck in the feature integration stage of object perception (Flom et al., 1963; He et al., 1996; Intriligator & Cavanagh, 2001; Levi & Waugh, 1994; Pelli et al., 2004; Pelli & Tillman, 2008). However, a recent model has proposed that object-level information can survive even under crowding, and the crowding effect is caused by limited access to conscious percepts, instead of the destruction of object representations (Chaney et al., 2014; Manassi & Whitney, 2018), which has been empirically supported (Fischer & Whitney, 2011; Kouider et al., 2011). Fischer and Whitney (2011) presented groups of faces to the left and the right peripheral visual fields and asked participants to judge the group of faces that had stronger emotional expressions. They found that the intensity of facial expressions at the center of the groups modulated the estimated average expression intensity, even when the central face was suppressed by crowding, suggesting that the visual system could access information on the emotional valence of facial expressions under crowding. If facial expressions can be processed even under crowded conditions, moral information, a crucial social signal, could be identified from faces in crowds. Therefore, it is crucial to clarify whether morality-related information modulates early perceptual stages to understand the mechanisms of perceptual and cognitive systems behind intuitive moral judgments.

We used a modified version of the affective learning task (Anderson et al., 2011), in which facial images were associated with moral valence. We presented participants with a structurally neutral face paired with a sentence describing an immoral or a neutral episode and instructed the participants to imagine the person with the face performing the act described in the sentence. In the crowding task that followed, we presented faces with immoral or neutral associations peripherally to the right or the left of a display and asked the participants to judge the morality or the gender of the target face. We expected both types of judgments to be less accurate when the target face was surrounded by other faces, compared to when it was presented alone (i.e., the crowding effect). Moreover, we predicted that both gender and moral judgments would be more accurate for immoral faces than for neutral faces if the immorality of a face facilitated the facial identification process under crowding. Alternatively, if information about morality does not facilitate the visibility of the faces, but information about immorality can be extracted in crowds, then the accuracy of moral judgments under crowded conditions would be higher for immoral faces than for neutral faces. However, the accuracy of gender judgments would remain unchanged.

## Experiment 1

### Method

#### Participants

Graduate and undergraduate students from Kwansei Gakuin University (*N* = 16, 8 men and 8 women, mean age = 20.19 years old) having a normal or corrected-to-normal vision participated in this study. All the participants provided their informed consent before participating in the study. We conducted a power analysis in advance to determine the minimum sample size. We decided that a minimum of 16 participants was needed to achieve a power level of 0.80 if we adopted the medium effect size (*f*) of 0.25. The Kwansei Gakuin University Institutional Review Board for Behavioral Research with Human Participants granted ethical approval for all the experiments conducted in this study.

#### Stimuli

We selected four facial images from the Chicago face database (Ma et al., 2015). The selected images consisted of Asian faces of two women and two men having an approximate age range of 20 to 40 years. All the faces were converted to grayscale and adjusted for the mean intensity, and the root mean square contrast (RMS contrast) by using the SHINE toolbox (Willenbockel et al., 2010) in MATLAB R2015a (Mathworks, Natick, MA).

We developed two types of episodic sentences: one consisted of sentences describing immoral behaviors such as, “They concealed their income by depositing most of their company's funds in overseas banks, and as a result, the salaries that should have been paid to their employees were reduced,” and the other, sentences describing neutral acts without any moral valence such as, “They went shopping to buy T-shirts in different colors and asked the clerk if they had them in stock” (see the Appendix). Ten such immoral and 30 such neutral episodes were developed by referring to sentences used in previous studies (e.g., Anderson et al., 2011; Konishi et al., 2017). We divided these 40 episodes into four sets of 10 episodes. There were two types of sets: “immoral-episode sets” that included immoral episodes (5 immoral episodes and 5 neutral episodes) and “neutral-episode sets” that included only neutral episodes (10 neutral episodes).

**Figure 1.**
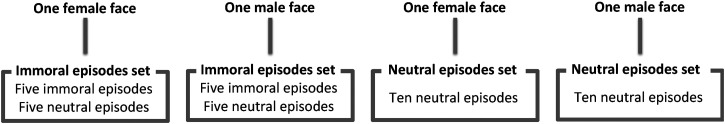
The assignment of faces and episodes. We prepared four sets of episodes and four faces.

We randomly divided the four faces into two sets consisting of one female and one male face that were paired with the immoral- or the neutral-episode sets such that two faces were paired with immoral-episode sets and the other two faces were paired with neutral-episode sets (see Figure 1). Faces were counterbalanced when pairings with immoral- or neutral-episodes sets across the participants.

Stimulus presentation was controlled by a Macintosh computer (Mac OS Sierra) equipped with MATLAB and Psychtoolbox programs. Stimuli were presented on a 24-inch LCD monitor (XL2420, BenQ) having a resolution of 1920 × 1080 pixels and a refresh rate of 100 Hz. Responses were made by using the “f” and “j” keys of the keyboard.

#### Procedures

**Figure 2.**
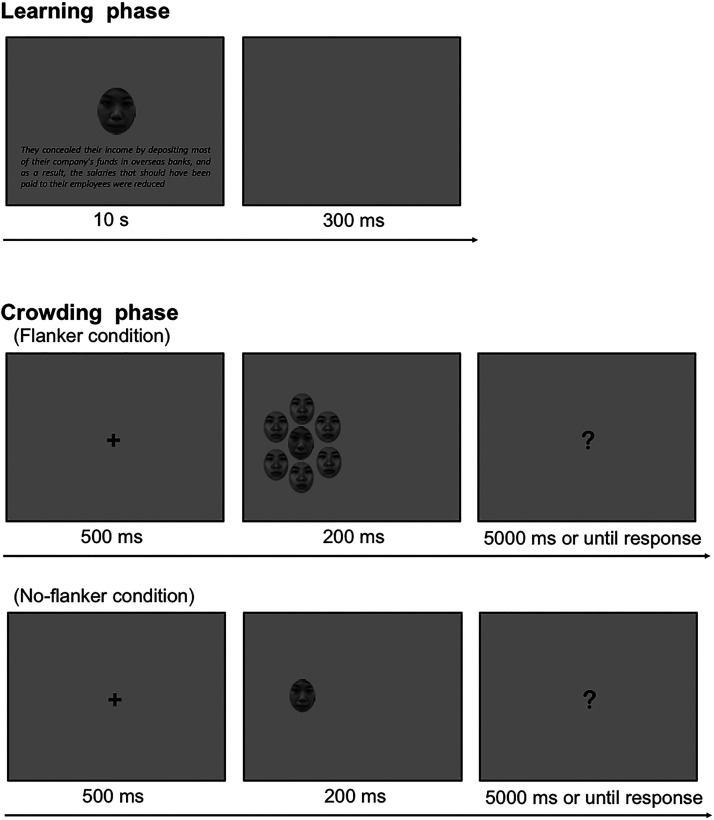
Crowding and learning phases procedures. The top figure shows the learning phase, and the bottom two figures show the crowding phase (with and without flankers). The target faces were the faces that were used in the learning phase. The flanker was an average face created by randomly selecting faces from the Chicago face database. The face is not the original image used in our experiments, but image used for illustrative purposes.

The experiment consisted of a learning phase and a crowding phase (see Figure 2). In the learning phase, the participants engaged in an imagination task, in which each trial started with presenting a sentence describing an episode (1° × 13°) and a facial image (1.82° × 2.42°). The participants imagined that the person with the presented face conducted the actions described in the episode and remembered whether the person was a bad person or not. The display was switched to a blank screen after 10 s, which remained for 300 ms, before, the next face and episode were presented. The pairs of faces and episodes were consistent throughout the experiment, and each pair was presented twice, such that the learning task included 160 trials. A recognition test was conducted after the participants completed the imagination task, in which the four facial images presented in the preceding imagination task appeared in random order. The participants were asked to judge whether the person in the facial image was a bad person (i.e., paired with an immoral episode) or not (i.e., paired with a neutral episode). The participants responded by pressing the “f” key of the keyboard if they judged the person was “a bad person” and the “j” if they judged that the person was “not a bad person.” The imagination task was repeated if a participant failed to correctly respond to all the recognition test trials, and the memory test was repeated until the recall accuracy reached 100%. The response-key mapping was counterbalanced across participants.

After the learning phase, the participants engaged in the crowding phase in which they conducted a judgment task. Each trial started by presenting a fixation cross (2.80° × 2.80°), and participants were instructed to maintain their gaze on the fixation cross. After 500 ms, a target face (1.49° × 3.08°) was presented for 200 ms, which was located at 6.00° eccentricity in the left or the right visual field and horizontally aligned to the fixation cross. The target face was either presented alone (no-flanker condition) or amongst six flankers (flanker condition) at a 50% probability. The distance from the center of the target face to the center of each flanker was set at 1.49°. The target faces presented in the crowding phase were the identical faces presented in the imagination task, and the faces of “a bad person” were named the immoral condition. Those of a “not a bad person” were named the neutral condition.

The crowding phase was divided into 2 blocks in which the participants performed different judgment tasks, either a gender judgment or a moral judgment task. The participants discriminated against the gender of the target face in the gender judgment task, whereas in the moral judgment task, they determined whether the target face had been paired with an immoral or a neutral episode. There were 320 trials in each block resulting in 640 trials. The key-response mapping and the order of the blocks were counterbalanced across the participants.

### Results

**Figure 3.**
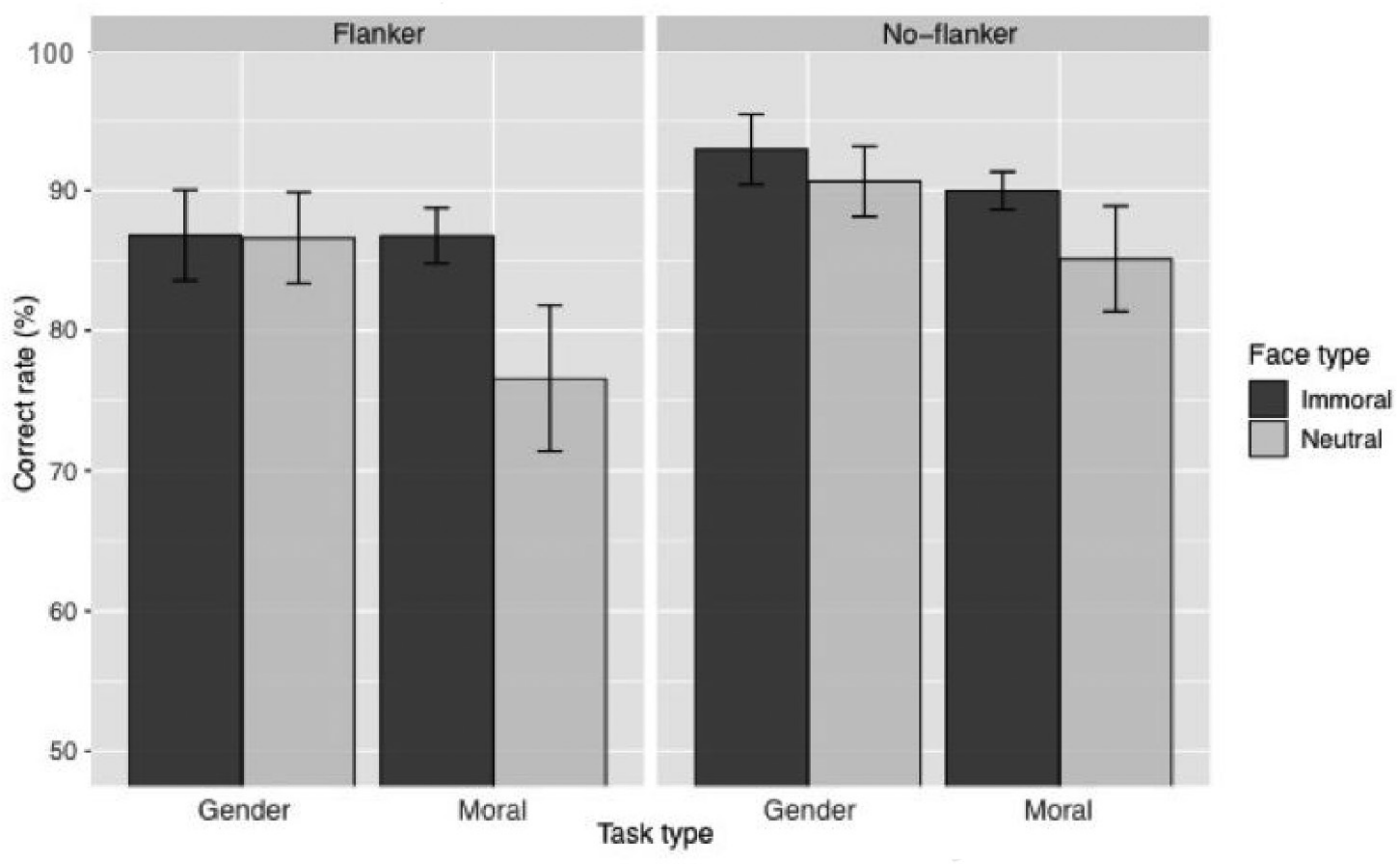
Mean of accuracy for each face type per judgment and flanker types. The error bar shows the standard error of the mean (*SEM*).

Figure 3 shows the accuracy of each judgment task and the flanker condition. It can be seen from the figure that the accuracy of gender and morality judgments are lower in the flanker condition than in the no-flanker condition and that the accuracy of moral judgments is higher for immoral faces than for neutral faces when the target face was flanked. We examined whether the type of face (immoral or neutral) influenced the increase in judgment accuracy by using a generalized linear mixed model for task type (gender or morality task) and the flanker condition (flanker or no-flanker condition) in which the participants were a random variable. Results indicated that the distribution of accuracy had a binomial distribution. We coded the face, task, and flanker type as 0.5/−0.5 (i.e., target face type: immoral = 0.5, neutral = −0.5, task type: gender judgment = 0.5, moral judgment = −0.5, flanker type: flanker = 0.5, no-flanker = −0.5). The free statistical software, R (version 3.5. 1; R Core Team, 2013), was used for data analysis. The generalized linear mixed model uses the glmer function, which is contained in the lme4 package (Bates et al., 2015).

Table 1 shows estimates and relative importance values. It can be seen from the table that the accuracy of gender judgments is higher than the accuracy of moral judgment (*b* = −0.45, *SE* = 0.06, *z* = −6.87, *p* < .001) and the flanker condition had a significant effect on the accuracy of gender and morality judgments (gender: *b* = −0.50, *SE* = 0.10, *z* = −6.16, *p* < .001; morality: *b* = −0.49, *SE* = 0.09, *z* = −5.78, *p* < .001), suggesting that crowding by flankers impaired gender and moral judgments. The face type predicted the accuracy of gender judgments in the no-flanker condition (*b* = 0.49, *SE* = 0.13, *z* = 2.23, *p* < .001); however, it did not predict the accuracy of gender judgments in the flanker condition (*b* = 0.02, *SE* = 0.12, *z* = 0.13, *p* = .50). Moreover, the face type predicted the accuracy of moral judgments in flanker and no-flanker conditions (flanker: *b* = 0.78, *SE* = 0.11, *z* = 6.95, *p* < .001; no-flanker: *b* = 0.34, *SE* = 0.15, *z* = 2.23, *p* = .03), suggesting that immorality could be identified even when the identification of a target face was impaired by crowding.

**Table 1.** Model-based estimates and relative importance values of each parameter.

glmer (Formula = Answer (0/1) ∼ Judgment type/flanker type/face type + (1| participants), family = binomial)										
Paramaters			*b*	*SE*	*z*	*p*		Effect size (d)	95% CI	
Intercept			2.24	0.23	9.88	< .001	***			
Judgment type			−0.45	0.06	−6.87	< .001	***	−0.50	−0.57	−0.32
Gender judgment	Flanker type		−0.60	0.10	−6.16	< .001	***	−0.68	−0.80	−0.41
Moral judgment	Flanker type		−0.49	0.09	−5.78	< .001	***	−0.55	−0.66	−0.33
Gender judgment	No-flanker condition	Face type	0.34	0.15	2.23	.03	*	0.38	0.04	0.64
Moral judgment	No-flanker condition	Face type	0.49	0.13	3.87	< .001	***	0.56	0.24	0.75
Gender judgment	Flanker condition	Face type	0.02	0.12	0.13	.90		0.02	−0.23	0.26
Moral judgment	Flanker condition	Face type	0.78	0.11	6.93	< .001	***	0.87	0.56	1.00

*Note.* SE means standard error of estimate. *** *p* < .001, * *p* < .05

The possibility remains, however, that the improved accuracy in identifying immoral faces when making moral judgments was caused by a response bias such that participants tended to respond with “a bad person” when they were unsure if they had seen a bad person or not. To examine whether the improved accuracy was explained only by a response bias or not, we computed the probability of hit responses (i.e., responding to an immoral face with “bad person”) and false alarms (i.e., responding to a neutral face with “bad person”), which were converted to indices of response bias (*β*) and sensitivity (*d’*) by using psycho packages in R (Makowski, 2018). A *β* value less than 1 indicated a bias towards responding with “bad person,” whereas a *β* value greater than 1 indicated a bias toward responding with “not bad person.” Moreover, a *d’* value of 0 indicated the inability to distinguish “immoral” signals from noise, whereas a *d’* value larger than 0 indicated an increased ability to distinguish “immoral” signals from noise. Analyzing the results of the no-flanker condition indicated a *β* value over 1 (*β* *=* 1.20; *t*(15) = 1.05, *p* = .31) and a *d’* value over 0 (*d’* *=* 2.48; *t*(15) = 11.99, *p* < .001), suggesting that the participants did not have a bias towards responding with “bad person,” but were able to detect immoral faces. Importantly, the results of the flanker condition showed a *β* value less than 1 (*β* *=* 0.88; *t*(15) = −2.20, *p* = .04), suggesting that participants tended to judge crowded faces as immoral. Moreover, a *d’* value over 0 (*d’* *=* 1.97; *t*(15) = 8.15, *p* < .001) suggested that participants’ sensitivity to immoral faces was significantly high even when the target face was flanked. These findings indicate that the accuracy was not susceptible to only a bias towards responding with “bad person.” We concluded that a high sensitivity to the morality of faces also contributed to the improved accuracy when making moral judgments for the immoral faces.

### Discussion

Experiment 1 investigated if the morality of faces could be detected automatically even when crowding impaired conscious percepts of the face. The results indicated that the judgment accuracy of the morality of immoral faces was higher than that of the morality of neutral faces. Moreover, the increased accuracy of moral judgments regarding immoral faces could not be explained just by response bias. These findings suggest that the morality of faces could be identified even when the target face was unclear, supporting previous findings that the emotional content of facial expressions is maintained under crowding, which biases subsequent perceptual judgments (Fischer & Whitney, 2011; Manassi & Whitney, 2018). In contrast to previous studies, the faces used in the current study had no emotional expressions. Therefore, our findings show that social information in faces that had no emotional reactions could be extracted under visual crowding.

## Experiment 2

Experiment 1 demonstrated that participants could process information about morality even under less discernible visual conditions. We designed Experiment 2 to investigate further whether information about morality could facilitate face identification under crowding. Specifically, the participants were instructed to choose the target face from two faces presented in the crowding phase with identical information about the morality of the two faces (i.e., two immoral or two neutral faces) or with different information (i.e., immoral and neutral faces). If information about the morality of faces could be extracted under crowding and used as a cue for selecting the target face, we expected that the accuracy of selecting a target face from different types of faces (i.e., immoral and neutral) would be higher when crowded faces are associated with immoral than neutral faces. Moreover, we expected that the accuracy of choosing among the same types of faces would be higher when an immoral face than a neutral face was presented in the crowding phase if information about morality facilitated processing detailed facial structures and improved face discrimination sensitivity.

Experiment 1 concluded that participants detected information about morality even under crowding because discrimination accuracy for faces associated with immoral episodes was higher than those associated with neutral episodes. However, an alternative explanation of this finding is that the association with a neutral episode or repeated presentations in the learning phase affected task performance. Experiment 2 examined these possibilities by comparing the crowding effect with faces associated with neutral information and “new faces” that neither appeared in the learning phase nor were associated with any episodes. If association with a neutral episode impairs perception under crowding, we would expect discrimination performance for neutral faces to be less accurate than for new faces. Alternatively, the increased familiarity of the face resulting from repetitive exposure in the learning phase modulated the crowding effect. If that were the case, we expected that discrimination performance would be higher for neutral than new faces.

### Method

#### Participants

Graduate and undergraduate students from Waseda University (*N* = 12, 6 men and 6 women, mean age = 20.92 years old) with normal or corrected-to-normal vision participated in Experiment 2. All the participants gave their informed consent before participating in the study. We conducted an advanced power analysis to determine the minimum sample size and decided that we needed a minimum of 9 participants to achieve a power of 0.80 if we adopted a medium effect size (*d*) of 0.45. We finally recruited 12 participants to counterbalance the task order and key-response mappings. Ethical approval for this study was obtained from the Ethics Review Committee on Research with Human Subjects at Waseda University.

#### Stimuli

We selected six facial images from the Chicago face database (Ma et al., 2015). The selected images comprised the faces of six Asian men. All the faces were converted to grayscale and adjusted for mean intensity and root mean square contrast (RMS contrast) by using the SHINE toolbox (Willenbockel et al., 2010) in MATLAB R2015a (Mathworks, Natick, MA).

We used two immoral- and two neutral-episode sets identical to Experiment 1. Then, we paired two faces with immoral-episode sets and two faces with neutral-episode sets. The other two faces were not paired with either set, and we only presented them in the crowding phase.

The presentation of all stimuli was controlled by a Macintosh computer (Mac OS Sierra) equipped with MATLAB and Psychtoolbox programs. Stimuli were displayed on a 23.5-inch LCD monitor (FG2421, EIZO), having a resolution of 1920 × 1080 pixels and a refresh rate of 100 Hz. Responses were made by pressing the “y” or “b” keyboard keys.

#### Procedures

Similar to Experiment 1, Experiment 2 consisted of a learning phase and a crowding phase. The learning phase was conducted using the identical procedure as in Experiment 1. The procedure of the crowding phase was identical to Experiment 1 except that the two faces appeared vertically after presenting the target in the crowding phase. Participants were asked to choose if the upper or the lower face was the target.

The crowding phase was divided into two blocks in which the presented target face was different in the morality block and the familiarity block. In the morality block, the target face in the crowding phase was identical to the faces presented in the learning phase. The immoral condition included faces of “a bad person,” whereas the neutral condition included faces of “not a bad person.” Pairs of two faces were divided into the same-type and different-type conditions, such that both faces were the same type as the target face in the same-type condition (i.e., both faces are “a bad person” or “not a bad person”). In contrast, the types of faces were different in the different-type conditions (i.e., a face of “a bad person” and a face of “not a bad person”). In familiarity blocks, the target faces in the crowding phase were slightly different from the faces presented in the learning phase. Moreover, the face of the “not a bad person” was named the neutral condition, whereas the new faces that were not presented in the learning phase were named the “new condition.” The two faces in the same-type condition of the new face block were new faces or “not a bad person.” A face of “not a bad person” and a new face were paired in the different-type conditions of the new face block. There were 320 trials in each block, resulting in 640 trials. The order of the blocks was counterbalanced across participants.

### Results

**Figure 4.**
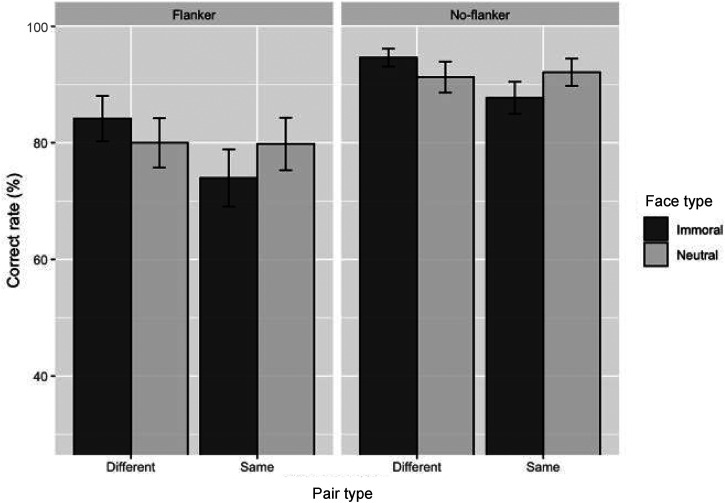
Mean accuracy of the morality block. Error bars show the standard error of the mean (*SEM*).

**Figure 5.**
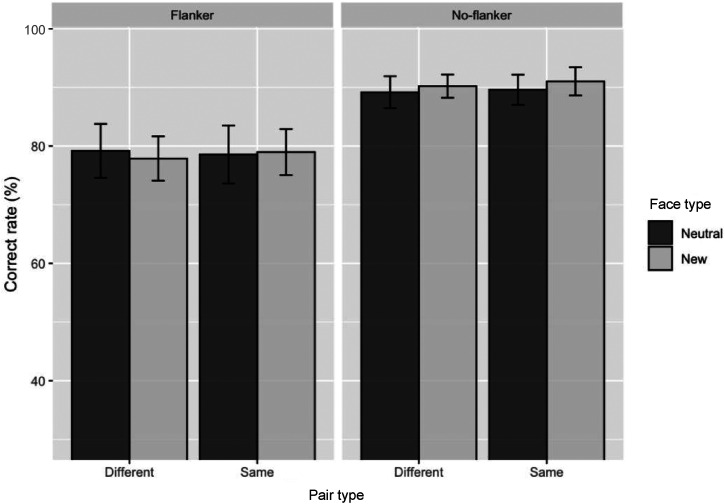
Mean accuracy of the familiarity block. Error bars show the standard error of the mean (*SEM*).

Figures 4 and 5 show the accuracy of choosing the target face from two faces. A visual inspection indicated that the accuracy is lower in the flanker than in the no-flanker condition. We examined whether the type of face (immoral or neutral) influenced the increase in accuracy by using a generalized linear mixed model for the type of face pairs (different-type or same-type) and the flanker condition (flanker or no-flanker condition) with the participants as a random variable. Results indicated that the accuracy distribution had a binomial distribution. We coded the face, pair, and flanker type as 0.5/−0.5 (i.e., target face type: immoral = 0.5, neutral = −0.5, pair type: different = 0.5, same = −0.5, flanker type: flanker = 0.5, no-flanker = −0.5). The free statistical software, R (version 3.5. 1; R Core Team, 2013), was used for data analysis. The generalized linear mixed model uses the glmer function contained in the lme4 package (Bates et al., 2015).

#### Morality Block

Table 2 shows estimates and relative significance values of the morality block. Results showed that the accuracy of the flanker condition was lower than the no-flanker condition (*b* = −1.09, *SE* = 0.10, *z* = −10.43, *p* < .001), suggesting that crowding by flankers impaired facial judgments. Moreover, the accuracy in the different-type condition was higher than the same-type condition in the two flanker conditions (flanker condition: *b* = 0.34, *SE* = 0.12, *z* = 2.89, *p* < .01; no-flanker condition: *b* = 0.41, *SE* = 0.17, *z* = 2.37 *p* = .02), indicated that same type face judgments were more difficult than different type face judgments. Moreover, the target faces’ face type in the different-type condition predicted facial judgment accuracy in the no-flanker condition (*b* = 0.53, *SE* = 0.26, *z* = 2.03, *p* = .04), but not in the flanker condition (*b* = 0.30, *SE* = 0.17, *z* = 1.72, *p* = .08). However, the target faces’ face type in the same-type condition predicted facial judgment accuracy in the flanker and no-flanker conditions (flanker: *b* = -0.35, *SE* = 0.16, *z* = −2.22, *p* = .03; no-flanker: *b* = −0.51, *SE* = 0.22, *z* = −2.28, *p* = .02). These findings suggest that the targets could be detected more correctly from neutral than immoral pairs under crowding.

**Table 2.** Model-based estimates and relative significance values of parameters in morality blocks.

glmer (Formula = Answer (0/1) ∼Flanker type/pair type/face type + (1| participants), family = binomial)										
Paramaters			*B*	*SE*	*z*	*p*		Effect size (d)	95% CI	
Intercept			2.08	0.25	8.44	< .001	***			
Flanker			−1.09	0.10	−10.43	< .001	***	−1.32	−1.57	−0.60
Flanker condition	Pair type		0.34	0.12	2.89	< .01	**	0.41	0.11	0.57
No-flanker condition	Pair type		0.41	0.17	2.37	.02	*	0.49	0.07	0.74
Flanker condition	Same type condition	Face type	−0.35	0.16	−2.22	.03	*	−0.43	−0.66	−0.04
No-flanker condition	Same type condition	Face type	−0.51	0.22	−2.28	.02	*	−0.62	−0.94	−0.07
Flanker condition	Different type condition	Face type	0.30	0.17	1.72	.08		0.36	−0.04	0.64
No-flanker condition	Different type condition	Face type	0.53	0.26	2.03	.04	*	0.64	0.02	1.04

*Note.* SE = standard error of estimate. *** *p* < .001, ** *p* < .01, * *p* < .05

#### Familiarity Block

Table 3 shows estimates and relative significance values of the familiarity block. Results indicated that the flanker condition’s accuracy was lower than the no-flanker condition (*b* = −0.94, *SE* = 0.10, *z* = −9.75, *p* < .001), suggesting that crowding by flankers impaired facial judgments consistent with the familiar face block. However, neither the pair type nor the face type in the flanker conditions was significant effects. These findings suggest that neither neutral episodic associations nor repeated exposure to faces during the learning phase affected visual crowding.

**Table 3.** Model-based estimates and relative significance values of each parameter in the familiarity block.

glmer (Formula = Answer (0/1) ∼flanker type/pair type/face type + (1| participants), family = binomial)										
Paramaters			b	SE	z	P		Effect size (d)	95% CI	
Intercept			1.20	0.30	6.62	< .001	***			
Flanker			−0.94	0.10	−9.75	< .001	***	−0.93	−1.13	−0.75
Flanker condition	Pair type		−0.02	0.11	−0.13	.89		−0.02	−0.24	0.21
No-flanker condition	Pair type		−0.07	0.15	−0.48	.64		−0.07	−0.38	0.23
Flanker condition	Same type condition	Face type	0.03	0.16	0.16	.87		0.03	−0.29	0.35
No-flanker condition	Same type condition	Face type	0.17	0.22	0.77	.44		0.17	−0.26	0.61
Flanker condition	Different type condition	Face type	−0.08	0.16	−0.52	.61		−0.08	−0.40	0.23
No-flanker condition	Different type condition	Face type	0.11	0.22	0.53	.60		0.11	−0.31	0.54

*Note.* SE = standard error of estimate. *** *p* < .001, ** *p* < .01, * *p* < .05

### Discussion

Experiment 2 examined whether the morality of faces facilitated facial identification under less discernible visual conditions. The results showed that the discrimination accuracy of immoral faces was higher than neutral faces in the different-type faces trials. However, this difference was not significant. Furthermore, contrary to our predictions, the same-type face trials’ accuracy was lower when the target was chosen from two immoral than two neutral faces. These results suggest that the immorality of crowded faces impaired rather than facilitated face identification, which we have discussed in the General Discussion. In addition, neutral associations did not affect the accuracy of choosing the target face from two faces compared to no associations, suggesting that neither neutral episodic associations nor repeated exposure to faces during the learning phase affected visual crowding.

## General Discussion

The current study examined whether the morality of a face could be detected even under less optimal visual conditions such as crowding. Experiment 1 demonstrated that the accuracy of moral judgments of immoral faces was higher than that of neutral faces under crowding, indicating that the morality of faces could be detected automatically even when the faces were crowded. However, Experiment 2 showed that crowding effects did not differ significantly between target face types (i.e., immoral or neutral) when choosing target faces from different types of faces. Experiment 1 asked participants to respond about the morality of a target face, whereas Experiment 2 required them to identify the target face from two faces, for which information about the morality of faces might not be useful for identifying a face.

Contrary to our expectations, the accuracy for identifying target faces with immoral associations was lower than for neutral associations when selecting target faces among the same type of faces irrespective of crowding, which is seemingly inconsistent with Experiment 1. However, combining Experiments 1 and 2’s results allows us to speculate that the immorality or negative valence of faces extracted under less discernible visual conditions impaired the individual faces’ structural identification, resulting in choosing a target from immoral faces being more difficult than from neutral ones. Previous studies have suggested that detecting target stimuli is diminished if the target is presented for a short time after an emotional distractor (Most, Chun, Widders, & Zald, 2005; de Jong, Koster, van Wees, & Martens, 2010; Ihssen, Heim, & Keil, 2007; Maratos, 2011). In contrast to Experiment 2, moral judgment accuracy of immoral faces increased under crowding in Experiment 1, which might be due to information extracted about morality that allowed the participants to respond correctly because they did not have to identify faces in this task. The identification accuracy of immoral and neutral faces did not differ significantly in the different face trials in the crowding condition of Experiment 2, probably because information about morality identified under crowding did not function as a cue effectively enough to modulate the identification performance.

We can interpret the results of the no-flanker condition without crowding similarly. Overall, the effect of morality in the no-crowding condition appears to be greater than in the crowding condition, suggesting that morality information is identified even under crowding, but it may not have been completed. Interestingly, we found also that gender judgments were more accurate for immoral faces than for neutral faces in the absence of crowding. It is unclear why face identification is impeded by morality, whereas gender judgments are facilitated. There has also been debate about how perceived social categories, such as gender, relate to face identity. Bruce and Young (1986) demonstrated that the gender of unfamiliar faces is easily categorized and suggested that gender and identity are coded to some extent independently. In contrast, recent findings suggest that gender is an invariant feature of the face; therefore, gender and identity categorization are not processed in parallel but in an integrated manner (Goshen-Gottstein & Ganel, 2000; Haxby et al., 2000; Rossion, 2002; Zhao & Hayward, 2013). Future research is needed to understand how morality information affects processing various facial features.

One may argue that our results are inconsistent with previous studies showing no effect of morality on unconscious processing (e.g., Stein et al., 2017). There are two possible explanations for the discrepancy between this and previous studies. The first explanation concerns the differences in experimental paradigms between previous works and the current ones. The study by Stein et al. (2017) used continuous flash suppression (CFS), whereas the present study used crowding. It has been reported that CFS suppresses signals in the early visual systems, which disrupts activities related to the object and facial processing (Kouider et al., 2011; Lin & He, 2009). On the other hand, the recently developed crowding model and latest studies have suggested that crowding might not interfere with object processing (Chaney et al., 2014; Fischer & Whitney, 2011; Kouider et al., 2011; Manassi & Whitney, 2018). Therefore, the lack of awareness about faces associated with negative gossip observed in the study by Stein et al. might reflect the methodological difference between the crowding and CFS paradigms. The second explanation concerns the differential intensity of morality. Stein et al. presented only one immoral episode with each face to associate a moral value with the face, whereas the current study presented multiple immoral episodes to each face. Therefore, faces associated with multiple immoral episodes might be more uncomfortable than faces associated with one specific immoral episode. It is suggested that future studies should further investigate the extent to which methodological differences in the learning phase change impressions regarding faces and the effect of such changes on the unconscious processing of faces. Furthermore, the degree of the crowding effect observed in the current experiment was relatively small compared to previous studies (Fischer & Whitney, 2011; Yeh et al., 2012). Therefore, conscious access to face information might not be wholly suppressed by crowding. Future research needs to examine whether immoral information can be extracted from information about facial identity under more crowded conditions.

The immoral and neutral episodes used in this study differed on several dimensions other than the moral information dimension. For example, immoral episodes seem more negative or unusual than neutral ones. Therefore, the possibility that the reduction of the crowding effect for the immoral faces was caused by factors other than moral information cannot be ruled out. There has been debate on “moral perception” (Firestone & Scholl, 2016; Gantman & Bavel, 2015), whether perception is preferentially attuned to moral content. In order to argue for the presence of “moral perception,” it is a crucial issue whether the researcher has successfully manipulated the moral variable alone, keeping out the influence of other residual variables. It would not be easy to dissociate these because moral valence is complex emotions that consist of multiple factors. Previous studies have argued the possibility that morality could be categorized into several psychological foundations (e.g., three foundations including autonomy: Shweder et al., 1997; five foundations including harm/care: Haidt & Graham, 2007; Haidt & Joseph, 2004). Each moral foundation has been pointed out to be linked to different emotional categories (e.g., harm-anger and purity-disgust links) and various degrees of weirdness (Gray & Keeney, 2015). Thus, rather than studying morality as a single construct, future studies should examine what factors characterize each moral foundation and to what extent each of these factors influences initial perception.

In conclusion, we demonstrated that the human visual system could detect social values, such as a person's morality, even from visual representations limited by crowding. The ability of humans to quickly judge the morality of another person before they are completely identified could facilitate avoiding adverse events, including harm and deception. Further research is needed to clarify the mechanism of rapid identification of social information about others and how it affects visual perception and cognition.

## Appendix

A1: Identification numbers of images used

CFD-AM-244-222-N, CFD-AM-208-143-N, CFD-AF-243-170-N, CFD-AF-245-143-N

A2: List of used episodes[Table table4-20416695221104843]

**Table table4-20416695221104843:**

Immoral episodes set 1	Immoral episodes	· They concealed their income by depositing most of their company's funds in overseas banks, and as a result, the salaries that should have been paid to their employees were reduced. · They drove to work, knowing that they were still drunk from the previous night. · They felt that their child's crying was loud and hit the child on the head hard to discipline the child. · While walking, they saw a pigeon resting on the roadside and went out of their way to kick the pigeon. · They passed their mistake on to their subordinates, and the subordinates committed suicide because of the strong public feelings against them.
	Neutral episodes	· They went shopping to buy T-shirts in different colors and asked the clerk if they had them in stock. · They switched on the computer in a hurry because they got an urgent job to make a document and send data to a client’s company. · They came to the movie theater, and there was a movie they wanted to see, so they decided to buy a ticket and enjoy the movie. · It suddenly started raining on their way to work, and they didn't have an umbrella, so they bought a plastic umbrella at a convenience store. · They went to look for a book that they wanted to read, but the book was out of stock, so they ordered the book.
Immoral episodes set 2	Immoral episodes	· They were frustrated because their work didn't go well, and they kicked a statue of a Buddhist deity and removed its head when they passed in front of a nearby shrine to relieve their stress. · They took a video of a live cockroach being disassembled by a cutter and uploaded the video on YouTube. · They are engaged in funerals and secretly took pictures of dead bodies for fun before they were cremated. · They wanted to urinate when they were swimming and peed in the pool without bothering to go to the bathroom. · They wanted to poo when they were cooking, and after going to the bathroom, they started cooking without washing their hands and served the food to their friends.
	Neutral episodes	· They were watching TV at home, but they got tired of watching halfway, so they were spacing out. · They went to the library to borrow some books, and as soon as they found the book they wanted, they borrowed it and read it at home. · The coffee maker broke down, so they bought a new one at a store near their house. · It was just noon, so they decided to stop working and have lunch. · Yesterday was payday. Since they were already at the convenience store near the bank, they print out their account record in their bankbook.
Neutral episodes set 1		· They checked the call history on their cell phone and saw a call from a friend, called back after work, and enjoyed chatting for a while. · They found a wonderful picture in the museum, and since they were interested in drawing pictures, they asked a commentator nearby how to draw the pictures. · A new cafe opened in their neighborhood, so they dropped in on their day off and bought some delicious looking cookies that were being sold. · They went to the mountains near their house on their day off because they like hiking and took a rest in the woods and had lunch. · They make a box lunch in the morning to save money, and it takes time to make different dishes, so they get up early every morning and make them. · The doorbell rang, so they went to the door to unlock the door. · They went to work in the morning and took their seats, and poured coffee. · They wanted to rent a room, so they went to the apartment superintendent's room and got information about the apartment from the superintendent. · It was lunchtime, and they sat on the sofa and ate while reading the newspaper. · It was a holiday, and they enjoyed the evening watching TV and reading books.
Neutral episodes set 2		· They checked the weather forecast on the news and thought about what to wear based on the temperature. · They found a good bar on their way home from work, and they dropped in for dinner. · They found the restaurant that the taxi driver recommended. They ate at the restaurant with their friend. · Their phone rang early in the morning, and the noise woke them up, so they quickly picked up the phone. · They saw fish jump when they were on a fishing boat, so they wanted to see what kind of fish it was, and they looked around with their binoculars. · They found the information about a bar in Kobe when they were reading a book, and they went to this bar with their friend. · They thought that they hadn't been to the beauty salon for a long time, so they had an appointment. Because they used the coupon that their friend gave them, they got a haircut cheaply. · They vacuumed the room clean because it was dirty and prepared a cleaning tool because they decided to remove the mold in the bathroom. · They were at work, and they lowered the air conditioner's temperature a little because they couldn't stand the summer heat. · They got a summer greeting card from their friend and went to the post office to buy postcards and stamps to return the letter.
